# Olfactory Thresholds of the U.S. Population of Home-Dwelling Older Adults: Development and Validation of a Short, Reliable Measure

**DOI:** 10.1371/journal.pone.0118589

**Published:** 2015-03-13

**Authors:** David W. Kern, L. Philip Schumm, Kristen E. Wroblewski, Jayant M. Pinto, Thomas Hummel, Martha K. McClintock

**Affiliations:** 1 Institute for Mind and Biology and Department of Comparative Human Development, The University of Chicago, Chicago, IL, United States of America; 2 Department of Public Health Sciences, The University of Chicago, Chicago, IL, United States of America; 3 Section of Otolaryngology-Head and Neck Surgery, Department of Surgery, The University of Chicago, Chicago, IL, United States of America; 4 Interdisciplinary Center Smell & Taste, ENT Clinic, TU Dresden, Germany; University of Graz, AUSTRIA

## Abstract

Current methods of olfactory sensitivity testing are logistically challenging and therefore infeasible for use in in-home surveys and other field settings. We developed a fast, easy and reliable method of assessing olfactory thresholds, and used it in the first study of olfactory sensitivity in a nationally representative sample of U.S. home-dwelling older adults. We validated our method via computer simulation together with a model estimated from 590 normosmics. Simulated subjects were assigned *n*-butanol thresholds drawn from the estimated normosmic distribution and based on these and the model, we simulated administration of both the staircase and constant stimuli methods. Our results replicate both the correlation between the two methods and their reliability as previously reported by studies using human subjects. Further simulations evaluated the reliability of different constant stimuli protocols, varying both the range of dilutions and number of stimuli (6–16). Six appropriately chosen dilutions were sufficient for good reliability (0.67) in normosmic subjects. Finally, we applied our method to design a 5-minute, in-home assessment of older adults (National Social Life, Health and Aging Project, or NSHAP), which had comparable reliability (0.56), despite many subjects having estimated thresholds above the strongest dilution. Thus, testing with a fast, 6-item constant stimuli protocol is informative, and permits olfactory testing in previously inaccessible research settings.

## Introduction

Current standardized, validated olfactory tests to determine olfactory sensitivity using psychophysical measures require many repeated and varied dilution presentations depending on the subject’s sensitivity. A common method for evaluating olfactory sensitivity is the Sniffin’ Sticks *n*-butanol threshold test (Burghart GmbH, Wedel, Germany; see [1,2]). This test uses a 3-alternative, forced-choice, single staircase method to determine sensitivity [1,3]. The time required to administer the threshold test depends on a subject’s performance and may be prolonged, particularly for those with olfactory dysfunction. In particular, population-based epidemiological studies—often conducted in the home by interviewers without specialized training in administering psychophysical measures—impose constraints of time and resources that preclude the use of gold-standard measures of olfactory assessment such as the staircase method.

Successful efforts to shorten the Sniffin’ Sticks olfactory sensitivity protocol using the method of constant stimuli [4] have been made [5,6]. While these methods have advanced the field of olfactory testing, they still require presentation of at least 16 dilutions and are therefore not practical in most field or survey settings due to time constraints and both subject and interviewer burden.

### Sniffin’ Sticks Threshold Test

The Sniffin’ Sticks threshold test involves 16 distinct dilutions administered using the staircase method. The test begins at one of the two weakest dilutions (pen 15 or 16), chosen randomly. During each presentation three felt tip pens are offered to the subject: one pen contains the target odor (a specific dilution of *n*-butanol) while the other two are blank, with the relative position of the target rotated with each presentation. Subjects are asked to indicate which of the three pens contains the target odor. Each time a subject correctly detects the odor at a given dilution, that same dilution is presented a second time in case the original correct response was due to chance alone (as expected at least one third of the time). Following a second consecutive correct response at a given dilution, the test is made more difficult by presenting a weaker dilution that is half the strength of the previous one. If at any point the subject gives an incorrect response, the test is made easier and a stronger dilution that is twice the strength of the previous is presented. Each change from weaker to stronger or stronger to weaker is considered a “reversal.” The Sniffin’ Sticks staircase protocol is administered until 7 reversals are observed. The dilutions presented at each of the last 4 reversals are then averaged to estimate that subject’s threshold [1,3] (hereafter referred to as the staircase-estimated threshold). This method is accurate and reliable for subjects whose thresholds fall within the range of dilutions used, but also time and labor intensive which is prohibitive in many settings.

### Staircase Method vs. Method of Constant Stimuli

The staircase method is dynamic, requiring the experimenter (or interviewer) to modify which dilutions are presented based on the subject’s previous responses, and similar to other psychophysical methods relies on the subject’s response history to determine when the procedure ends [1,3,7,8]. Although yielding a reliable threshold estimate, the method is relatively complex and requires significant time (up to 20 minutes) and training to administer [1].

In contrast, the method of constant stimuli uses a fixed number of presentations. Each subject receives the same set of stimuli, though their order may be varied between subjects (ideally in a random manner) to avoid bias due to possible order effects. While this method is typically less efficient than adaptive methods (which administer a higher proportion of stimuli near each specific subject’s threshold), it is considerably easier to administer and, unlike the staircase method, administers the same total number of stimuli to each subject.

Lötsch and colleagues [6] demonstrated that thresholds obtained using the method of constant stimuli with 16 dilutions are strongly correlated with those obtained using the staircase method (r = 0.84). Further, the method of constant stimuli exhibited similarly good test-retest reliability (0.79 versus 0.82 for staircase). They administered the Sniffin’ Sticks threshold test utilizing the staircase method to 100 subjects using phenyl ethyl alcohol (PEA), another odorant commonly used in this staircase threshold test. To the same 100 subjects, they also administered a constant stimuli version of the threshold test using 16 dilutions. Each dilution was administered once in random order, and the threshold (defined as the dilution yielding a correct response probability of $\frac{2}{3}$) was estimated separately for each subject using an Item-response Theory (IRT) model (described below).

Lötsch et al. demonstrated that the method of constant stimuli can be applied to olfaction threshold testing yielding reliable validated data. Although shorter than the staircase method, testing 16 dilutions still requires the presentation of 48 separate pens (16 targets and 32 blanks). See also Wise et al. [9] who investigate two alternative methods for reducing the time required to measure olfactory sensitivity, both of which still require a relatively large total number of stimuli.

### Study Overview

Our goal was to design a shorter, easier olfactory threshold test suitable for survey studies and yet accurate and reliable enough for scientific research. Such a test would permit incorporating olfactory threshold assessment into existing large, population-based studies, thereby allowing olfaction research among both the general population and previously understudied subpopulations, as well as interdisciplinary research on the relationships between olfactory function and physical and mental health. We accomplished this goal in three steps: (1) We developed an IRT model for estimating olfactory thresholds using data from either the staircase or constant stimuli protocols, and validated it using previously collected staircase data from 590 normosmics; (2) We used computer simulations based on the model to replicate the results of a previous study in human subjects comparing staircase and constant stimuli protocols, and to examine the effects of reducing the number of constant stimuli (dilutions) used; and (3) We developed and administered a 6-dilution constant stimuli protocol to a representative sample of the home-dwelling, older adult population of the United States, and used the simulation-based method in (2) to evaluate the accuracy and reliability of the resulting data.

## Methods

### Item Response Theory (IRT) model

As described above, the staircase method is usually scored by averaging the dilutions corresponding to the last four reversals to estimate an individual’s true underlying threshold—an approach not available following the administration of constant stimuli. An alternative approach, applicable to data from either the staircase or constant stimuli method, is to fit a statistical model to the data from which an estimate of the true underlying threshold may be obtained. When used with staircase data, this approach is more efficient because it uses a subject’s entire response history (instead of just the last four reversals) to estimate his or her threshold.

A common model for such data is a logistic IRT model in which the probability of correctly identifying the odorant pen increases monotonically with increasing concentration, from a lower asymptote of $\frac{1}{3}$ (for concentrations that are undetectable by the subject and therefore equally likely to be selected as each of the two blank pens) to an upper asymptote of one (for concentrations that are always detected). This model may be written as

$$P\left(Y_{ij}=1\right)=\frac{1}{3}+\frac{2}{3}{\text{Logit}}^{-1}\left(\alpha_{i}-\beta d_{ij}\right)$$ (1)

where *Y_ij_* denotes the response (1 for correct or 0 for incorrect) by the *i*th subject for the *j*th presentation with dilution step *d_ij_*, *β* is a slope parameter describing the rate at which the probability of a correct response decreases with increasing dilution, *α_i_* is a subject-specific intercept describing the subject’s underlying olfactory ability, and Logit^−1^(↺) is the inverse logistic function. For the Sniffin’ Sticks threshold test, *d_ij_* ranges from 1 (4% solution) to 16 (4% × 1/2^15^ = 1.22 ppm). The model may be fit separately to each subject’s data (as in [6]) or simultaneously to the data from multiple subjects. The latter involves modeling the parameters *α_i_* as drawn from an underlying probability distribution (typically taken to be the Normal distribution); the resulting model is known as a Generalized Linear Latent and Mixed Model (GLLAMM) [10]. In the analyses reported below, this model was fit in Stata release 13 [11] using the gllamm package release date September 11, 2011 [12].

Setting *P*(*Y_ij_* = 1) to $\frac{2}{3}$ in Equation (1) and solving for *d_ij_* yields *α_i_* / *β*, which corresponds to the threshold for subject *i*. Thus, threshold estimates can be obtained after fitting the model by using estimates of each individual’s underlying olfactory ability (*α̂_i_*) (obtained, for example, via empirical Bayes methods) and the estimated slope (*β̂*). These *model-estimated thresholds* represent the point on the dilution-response curve corresponding to a $\frac{2}{3}$ probability of a correct response, and are therefore comparable to the staircase-estimated threshold, which estimates the same point on the dilution-response curve by taking the average of the last four reversals.

We fit the GLLAMM model described above to previously collected *n*-butanol Sniffin’ Sticks threshold data obtained via the staircase method from 590 normosmic subjects (all of whom were tested at the ENT-Department of the TU Dresden) [13]. Normosmic subjects were identified based on a cumulative “TDI score,” a summation of the scores of the Threshold task, a Detection task, and an Identification task [14].

### Examining the Properties of Alternative Protocols Using Computer Simulation

Lötsch et al. [6] demonstrated that constant stimuli administration of 16 dilutions may be used to shorten and simplify the assessment of olfactory threshold and still achieve high reliability, which raises several additional questions. Among these are: (1) Can the instrument be shortened further, for use in settings with stringent time and/or other constraints; (2) Given a fixed (possibly smaller) number of stimuli, what is the optimal subset of dilutions to choose relative to the distribution of olfactory abilities in the population being studied; and (3) Can one estimate the reliability of a given constant stimuli protocol without conducting a separate human subjects validation study?

To answer these questions, we developed software for simulating the administration of both the staircase method and the method of constant stimuli to a sample of virtual subjects with olfactory abilities drawn from a specified distribution. By using a distribution chosen to match that of a target population (ideally estimated from previously collected data or from the existing literature) and by varying the number and dilutions of the stimuli used, we can evaluate and compare the measurement properties of several constant stimuli designs. This procedure can thus be used both to design a new study and to evaluate the properties of protocols used in previous studies.

### Application to a Population-Based Study of Aging

The National Social Life, Health and Aging Project (NSHAP) is a U.S. national longitudinal study of health and aging, with baseline data collected from a probability sample of 3,005 adults aged 57–85 in 2005–6 (Wave 1). A follow-up including all respondents still living *and* their spouses/partners was conducted in 2010–11 (Wave 2). Waves 1 and 2 of NSHAP were approved by the Institutional Review Boards of The University of Chicago and the National Opinion Research Center (NORC); all respondents provided written, informed consent. The weighted distributions of demographic variables in the NSHAP sample closely match those from the U.S. 2002 Current Population Survey, confirming that the NSHAP sample is representative of the U.S. population of home-dwelling, older adults [15].

Odor identification was measured in Wave 1 [16,17]. At Wave 2 we again measured odor identification and added a threshold measurement to permit the first ever estimates of olfactory threshold for this population. Unfortunately, the omnibus nature of the study which includes a lengthy questionnaire, several physiological measurements and collection of several biological samples, all conducted in the home, precluded use of the staircase method. Specifically, the study did not allow the time required for the interviewers—who did not have training or experience in the administration of psychophysical measurements—to administer the staircase method or even a 16-dilution constant stimuli protocol, both of which would have exceeded the 5 minutes available. Further, the interviewers were limited in their ability to transport additional supplies to respondents’ homes, given all of the other materials necessary to complete the interview. Thus, we had to develop a shorter and simpler method of olfactory threshold that reduced both interviewer and respondent burden.

### NSHAP Threshold Protocol

Although there were no existing data on olfactory sensitivity in the U.S. population of older adults, based on previous threshold testing in this age group [13,18] and results from the odor identification data in Wave 1, we anticipated that the distribution of thresholds would have a considerably lower mean than for younger adults. Thus, we selected the following 6 dilutions of *n*-butanol to target the expected distribution of thresholds: 0.13%, 0.25%, 0.50%, 2.0%, 4.0% and 8.0%, which correspond to dilution steps 6, 5, 4, 2, 1 and 0 in the Sniffin’ Sticks threshold test. Although pen 1 (4% *n*-butanol) is the strongest dilution used as part of the Sniffin’ Sticks threshold test, given the expected prevalence of olfactory dysfunction in NSHAP’s older adult population, we added the 8% *n*-butanol pen (pen 0) into our protocol to enhance our ability to discriminate among those with dysfunction.

These target dilutions were each administered along with two additional blank pens, in order of increasing concentration (from pen 6 to pen 0) to minimize the possibility that a relatively strong concentration might subsequently make it more difficult to detect a weaker concentration (habituation) [19]. The position of the target pen as either the first, second, or third pen for a given presentation was varied across the six presentations, but uniform for all respondents. Although randomizing both the order in which the dilutions were administered and the position of the target pen would have been ideal, this additional complexity would have increased the time and the risk of administration error.

Unlike clinical or laboratory settings, in order to maintain cooperation and a good rapport throughout the interview, respondents in survey studies are typically permitted to refuse to answer any question, or to respond by indicating that they don’t know. Thus, although NSHAP interviewers were instructed to encourage respondents to do their best to select a pen even if they were not sure which was the target, some respondents still insisted that they didn’t know or refused to answer. Not surprisingly, such responses were more common at the weakest concentrations. Treating such responses as incorrect would create a negative bias in the threshold estimates, since had these respondents guessed, they would have been correct at least one-third of the time by chance. Thus, in order to obtain a more accurate estimate of the threshold distribution for use in the work described below, we used multiple imputation with an imputation model which specified that the probability of a correct response in these cases was $\frac{1}{3}$ [20]. This approach could also be used when generating estimates of individual thresholds.

## Results

### Validation of IRT Model Among Normosmics

**Fig 1:**
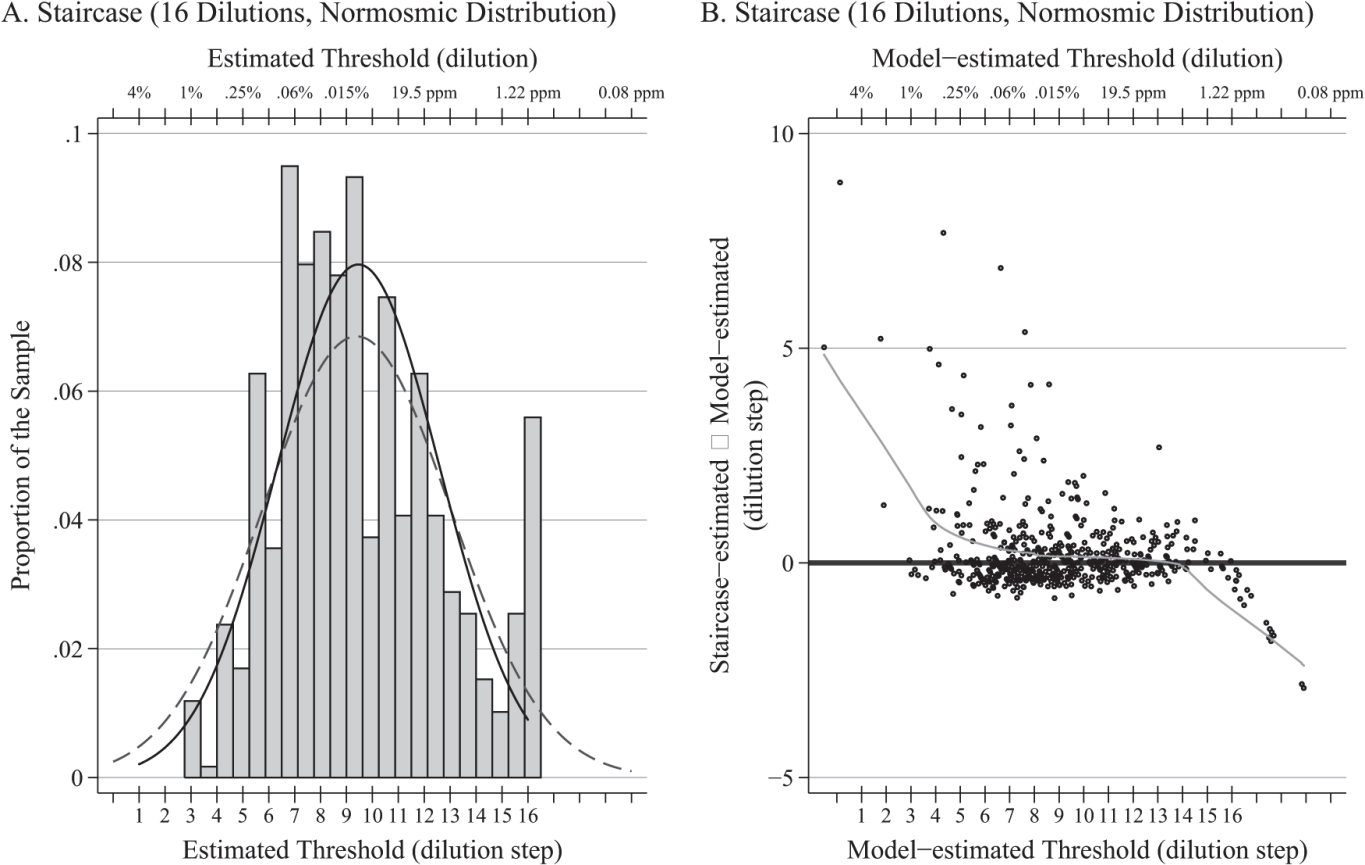
Histogram and Bland-Altman plot. **A.** Histogram of 590 normosmic staircase-estimated thresholds (as determined by the mean of the last 4 reversals), together with a normal density curve with the same mean and standard deviation (solid black). In addition, the distribution of estimated thresholds from the fitted model is also plotted (dashed grey).B. Corresponding Bland-Altman plot comparing staircase-estimated thresholds and model-estimated thresholds among the 590 normosmics together with a LOWESS smoother.

Among the normosmic sample, the estimated slope parameter (*β*) was 1.12 (SE = 0.06), and the mean and variance of the *α_i_* were estimated as 10.5 (SE = 0.55) and 16.6 (SE = 2.0), respectively. Fig. 1A shows a histogram of the 590 staircase-estimated thresholds determined from the average of the last four reversals, together with a Normal density curve with the same mean and standard deviation (solid black). In addition, the distribution of thresholds (obtained by dividing *α_i_* by *β̂*) estimated from the IRT model is also plotted (grey dashed). The means of the two distributions are nearly identical, demonstrating excellent correspondence between the model-estimated thresholds and threshold estimates obtained using the staircase method, thereby validating the model. As expected, the variance of the model-estimated distribution is slightly greater, because the staircase method does not permit threshold estimates outside of the range of dilutions administered (1–16).

Fig. 1B shows a Bland-Altman plot [21] comparing the two sets of estimates (difference between staircase-estimated and model-estimated thresholds versus the model-estimated threshold) together with a LOWESS smoother. For those in the middle 50% of the model-estimated threshold distribution (7.0–11.2), the average difference between staircase and model-estimated thresholds is only 0.16 with a standard deviation of 0.80, reflecting a relatively close correspondence between the two measures despite a small, positive bias in the staircase estimates.

At the high end of the distribution of model-estimated thresholds where the odorant concentrations are weakest, the staircase estimates demonstrate a clear negative bias. This is due to the fact that the maximum possible value of the staircase estimates is 16, even among those whose true threshold may be even higher. In contrast, at the low end of the distribution where the odorant concentrations are strongest, the staircase-estimated thresholds exhibit a positive bias. This is due to the fact that the staircase protocol begins at the weak end of the dilutions and works its way toward the strong end; erroneous reversals early on to a weaker dilution (reversing to a weaker dilution despite having a true threshold at a stronger dilution) can then result in a misleadingly weak dilution being included when computing the staircase-estimated threshold.

### Replicating Lötsch et al

To demonstrate that our computer simulation method yields similar results to those from a study using human subjects, we used our method to replicate the results of Lötsch et al. [6]. Following their protocol, we simulated the administration of both the staircase and constant stimuli methods with 16 dilutions to 100 subjects. Although Lötsch et al. utilized a general sample of adults including both normosmics *and* those with olfactory dysfunction (approximately 18%), their publication did not report an overall mean and standard deviation of the estimated thresholds, and thus we drew abilities for our simulated subjects from the distribution of abilities estimated from the 590 normosmics described above. Given these abilities, responses were then simulated from the model above using the estimated value of 1.12 for *β*. Five hundred replications were performed, each with a sample of 100 subjects.

Our simulations yielded mean reliabilities for the staircase and constant stimuli methods of 0.86 (not shown) and 0.84 (Table 1), respectively, only slightly higher than the test-retest reliabilities reported by Lötsch et al. of 0.82 and 0.79, and nearly identical to results reported by Haehner at al. [22] who estimated a test-retest reliability of 0.85 for the staircase method when administered to human subjects. In addition, the mean correlation between the staircase and constant stimuli methods was 0.86, as compared to 0.84 for Lötsch et al. Of note, Lötsch et al.’s reliability estimates were based on a single sample of 100 subjects, and may therefore reflect some random variation (compare the sampling variability represented by the 5^th^ and 95^th^ percentiles of the reliability distributions reported in Table 1) as opposed to our estimates obtained by averaging the results from 500 independent replications.

**Table 1:** Reliability of several constant stimuli protocols based on simulated administration to the 590 normosmic distribution, n = 100 subjects with 500 replications.

	Number of Stimuli	16	12	8	6	(6)x2^a^
**Evenly Distributed**	Dilutions Administered	1–16	1–3, 5–7, 9–11, 13–16	2–16 (Even)^b^	2, 5, 8, 9, 12, 15	(2, 5, 8, 9, 12, 15)x2
	Mean Reliability (SE)^c^	0.843 (0.001)	0.794 (0.002)	0.706 (0.003)	0.649 (0.003)	0.805 (0.002)
	5%, 50%, 95%	0.789, 0.844, 0.889	0.720, 0.797, 0.850	0.607, 0.711, 0.787	0.538, 0.657, 0.741	0.739, 0.809, 0.859
	% of Convergence Failures	0.2%	2.6%	29.8%	24.6%	0.6%
**Centered**	Dilutions Administered	NA^d^	5–16	7–14	8–13	(8–13)x2
	Mean Reliability (SE)		0.805 (0.002)	0.732 (0.002)	0.660 (0.002)	0.776 (0.002)
	5%, 50%, 95%		0.739, 0.807, 0.863	0.657, 0.736, 0.801	0.568, 0.668, 0.736	0.712, 0.781, 0.827
	% of Convergence Failures		1.4%	1.2%	8.2%	0.0%
**Tails**	Dilutions Administered	NA	1–6, 11–16	5–8, 13–16	5–7, 14–16	(5–7, 14–16)x2
	Mean Reliability (SE)		0.719 (0.003)	0.684 (0.002)	0.551 (0.003)	0.723 (0.002)
	5%, 50%, 95%		0.616, 0.725, 0.802	0.586, 0.687, 0.770	0.435, 0.550, 0.660	0.645, 0.726, 0.800
	% of Convergence Failures		6.0%	11.8%	29.2%	1.2%
**Shifted**	Dilutions Administered	NA	1–12	1, 3, 5, 7–11	1, 3, 5, 7, 9, 11	(1, 3, 5, 7, 9, 11)x2
	Mean Reliability (SE)		0.795 (0.002)	0.732 (0.002)	0.642 (0.002)	0.783 (0.002)
	5%, 50%, 95%		0.735, 0.798, 0.853	0.658, 0.735, 0.798	0.551, 0.645, 0.730	0.715, 0.789, 0.836
	% of Convergence Failures		2.4%	10.0%	29.2%	0.8%

^a^This configuration presents the identical 6 dilutions used for a given protocol twice for a total of 12 stimuli

^b^A configuration using 8 odd dilutions yielded similar results

^c^SE = standard error

^d^NA = not applicable.

Our reliability estimates may also be slightly higher than their test-retest reliability estimates due to one or more of the following: (1) variability across days in both the administrator and the subject that is not captured in our simulation (though prior work has shown that the Sniffin’ Sticks are reliable with repeated testing and across days [23]), (2) possible variation in effective pen concentrations with repeated use, also not captured in our simulation (though a recent publication [24] has demonstrated that this effect is relatively small), and (3) potential differences in reliability between PEA and *n*-butanol (though a publication by Croy et al. [25] suggests that these odors exhibit similar reliability). In light of these differences, our results are quite consistent with those of Lötsch et al., demonstrating the ability of our method to reproduce reliability estimates from studies of human subjects.

### Reducing the Number of Stimuli

Next, we used our method to examine the effects of reducing the number of stimuli and of the choice of dilutions relative to the threshold distribution of the population. We evaluated designs using 12, 8, and 6 stimuli, carefully selecting their dilutions to cover the range of thresholds, as well as targeting specific areas of the distribution. Like before, a sample size of 100 subjects with 500 replications was used.

As expected, reducing the number of stimuli reduced the reliability from 0.79 for 12 evenly distributed dilutions down to 0.65 for only 6 dilutions (Table 1). This was due entirely to reducing the number of stimuli presented (as opposed to reducing the number of distinct dilutions), since presenting 6 dilutions each twice (12 stimuli total) yielded nearly identical reliability (0.81) as 12 distinct dilutions. Interestingly, the reliability is relatively robust to differences in the selection of dilutions, such as using a smaller range of dilutions under the center of the ability distribution or shifting the dilutions to one end of the distribution. Only the scenario in which the dilutions were located exclusively at the tails of the distribution (with none in the center) yielded a significant decrease in reliability.

Although data from 16 dilutions can be analyzed by fitting the model separately to each subject’s data (as done by Lötsch et al.), a smaller number of dilutions requires using a random effects model as we have done here. Still, as seen in Table 1, we were unable to obtain threshold estimates in a substantial fraction of replications when using 8 or fewer stimuli, due to the model’s failure to converge. This problem was nearly eliminated in all but one case (Tails) by moving to a sample size of 1,000 subjects (Table 2). Similar convergence problems could be avoided in smaller studies by using Bayesian methods to fit the model, ideally basing the priors on existing data [26].

**Table 2:** Reliability of several constant stimuli protocols based on simulated administration to the 590 normosmic distribution, n = 1,000 subjects with 500 replications.

	Number of Stimuli	16	12	8	6	(6)x2^a^
**Evenly Distributed**	Dilutions Administered	1–16	1–3, 5–7, 9–11, 13–16	2–16 (Even)^b^	2, 5, 8, 9, 12, 15	(2, 5, 8, 9, 12, 15)x2
	Mean Reliability^c^	0.844	0.797	0.709	0.651	0.806
	5%, 50%, 95%	0.828, 0.845, 0.860	0.775, 0.796, 0.817	0.682, 0.709, 0.736	0.616, 0.652, 0.680	0.787, 0.806, 0.825
	% of Convergence Failures	0.0%	0.0%	6.6%	4.2%	0.0%
**Centered**	Dilutions Administered	NA^d^	5–16	7–14	8–13	(8–13)x2
	Mean Reliability		0.809	0.732	0.665	0.772
	5%, 50%, 95%		0.791, 0.809, 0.826	0.708, 0.731, 0.755	0.637, 0.664, 0.694	0.753, 0.772, 0.791
	% of Convergence Failures		0.0%	0.0%	0.0%	0.0%
**Tails**	Dilutions Administered	NA	1–6, 11–16	5–8, 13–16	5–7, 14–16	(5–7, 14–16)x2
	Mean Reliability		0.727	0.687	0.555	0.725
	5%, 50%, 95%		0.697, 0.727, 0.754	0.662, 0.688, 0.713	0.507, 0.558, 0.597	0.698, 0.725, 0.750
	% of Convergence Failures		0.0%	0.0%	31.8%	0.0%
**Shifted**	Dilutions Administered	NA	1–12	1, 3, 5, 7–11	1, 3, 5, 7, 9, 11	(1, 3, 5, 7, 9, 11)x2
	Mean Reliability		0.796	0.733	0.644	0.783
	5%, 50%, 95%		0.775, 0.796, 0.815	0.709, 0.733, 0.755	0.614, 0.644, 0.672	0.761, 0.783, 0.803
	% of Convergence Failures		0.0%	0.0%	4.2%	0.0%

^a^This configuration presents the identical 6 dilutions used for a given protocol twice for a total of 12 stimuli

^b^A configuration using 8 odd dilutions yielded similar results

^c^All reliabilities have a standard error ≤ 0.001

^d^NA = not applicable.

**Fig 2:**
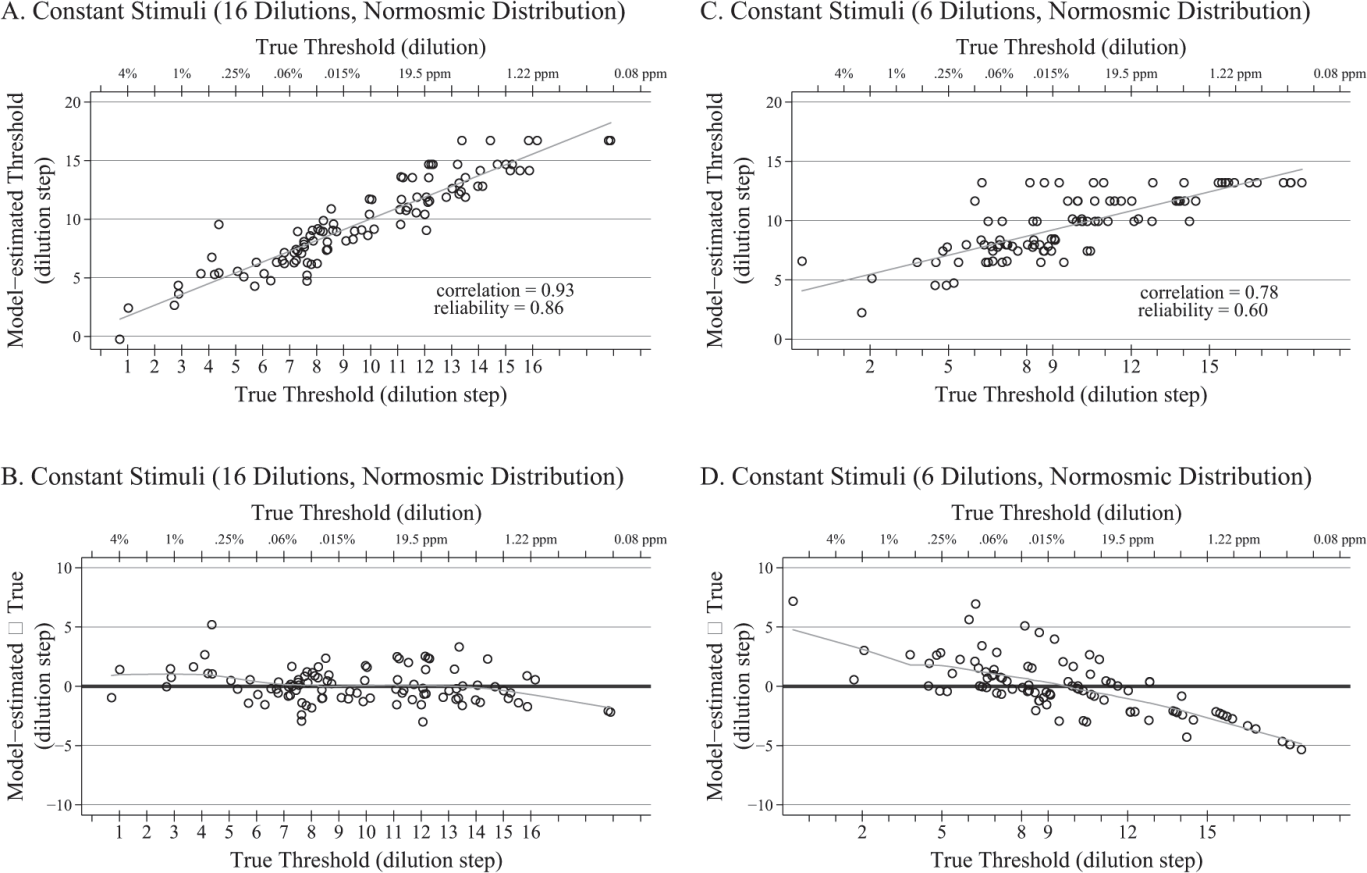
Scatter and Bland-Altman Plots for Normosmics. A. Scatter plot (plus regression line) of the relationship between the model-estimated thresholds and the true thresholds for a single replication of the 16-dilution constant stimuli design, with thresholds drawn from 590 normosmic distribution (n = 100). B. Corresponding Bland-Altman plot with LOWESS smoother of the 16-dilution constant stimuli design, with thresholds drawn from 590 normosmic distribution (n = 100). C. Scatter plot (plus regression line) of the relationship between the model-estimated thresholds and the true thresholds for a single replication of the 6-dilution constant stimuli design, with thresholds drawn from 590 normosmic distribution (n = 100). The 6 dilutions are evenly distributed across the range of possible thresholds and the administered dilution steps are noted on the lower x-axis. D. Corresponding Bland-Altman plot with LOWESS smoother of the 6-dilution (evenly distributed) constant stimuli design, with thresholds drawn from 590 normosmic distribution (n = 100).

A plot showing the relationship between the model-estimated threshold and the true threshold for a single replication (n = 100) of both the full 16-dilution design and the 6-dilution design (with dilutions evenly distributed over the range of dilutions) is shown in Fig. 2 (Panels A and C). Corresponding Bland-Altman plots (model-estimated threshold minus true threshold plotted versus true threshold) depicting the difference between the model-estimated and true threshold together with a LOWESS smoother are also shown in Fig. 2 (Panels B and D). The correlation between the model-estimated and true thresholds for the 6-dilution design is 0.78 for this replication, only slightly below the average of 0.81 across all replications. The biggest difference between the two designs is the conditional bias (i.e., conditional on the true threshold) for the 6-dilution design, which increases toward the tails of the distribution. This conditional bias is a property of the empirical Bayes method we have used to estimate the individual thresholds, and is due to the individual estimates being shrunk toward the overall mean. The amount of shrinkage decreases as the reliability of the estimates increases; it is, for example, substantially reduced if each of the 6 dilutions is presented twice. Despite this, the empirical Bayes estimates are unconditionally unbiased, thereby permitting estimation of the mean threshold for the population [27].

Importantly, this shrinkage is beneficial when using the estimated thresholds as a covariate in the analysis of other variables. Consider an analysis examining the relationship between olfaction and another health outcome (for example cognitive function) in which the outcome is regressed on olfaction threshold, perhaps adjusting for additional covariates. If threshold estimates obtained using the average of the last four reversals (as in the staircase method) or by fitting an IRT separately to the data for each individual (as in Lötsch et al.) are used, the estimated slope will be biased downward in magnitude, with the amount of bias increasing as the reliability decreases. This well-known phenomenon is known as *regression attenuation* or *dilution*. However, using the empirical Bayes estimates to measure olfactory threshold can eliminate this bias [28]. As noted above, this advantage can be achieved regardless of whether the data are collected using the staircase or constant stimuli methods.

### Estimates of Threshold for the U.S. Population of Older Adults

Two-thousand two-hundred and seven NSHAP Wave 2 respondents, aged 62–90 at the time of the interview, completed the 6-dilution *n*-butanol constant stimuli detection task. Comments from field interviewers indicated that respondents reacted favorably to the olfactory protocol [29].

As expected, the estimated mean threshold for older adults in the U.S. was substantially lower than for the normosmic sample (2.4 vs. 9.4), while the estimated standard deviation was only slightly smaller (3.0 vs. 3.6). The estimated slope parameter was 0.76, indicating that the probability of a correct response increases less quickly with increasing concentration than for the normosmics (for whom the slope was estimated to be 1.12). This is due to the fact that some respondents in this older adult sample may be completely anosmic, and therefore for them, an increase in concentration does not increase the likelihood of a correct response. Although this could be accommodated by extending the model, fitting such a model would require more extensive data than are available here (see discussion).

### Accuracy and Reliability of the NSHAP Protocol

To evaluate the measurement properties of the NSHAP protocol, we performed a computer simulation study in the same manner as described above. Although the GLLAMM model fit to the NSHAP data assumes a Normal distribution of olfactory thresholds, the estimated Normal distribution with mean 2.4 and standard deviation 3.0 corresponds to 12% of the population having a threshold of 16% *n*-butanol (pen-1) or stronger and 7% of the population having a threshold of 32% *n*-butanol (pen-2) or stronger.

**Fig 3:**
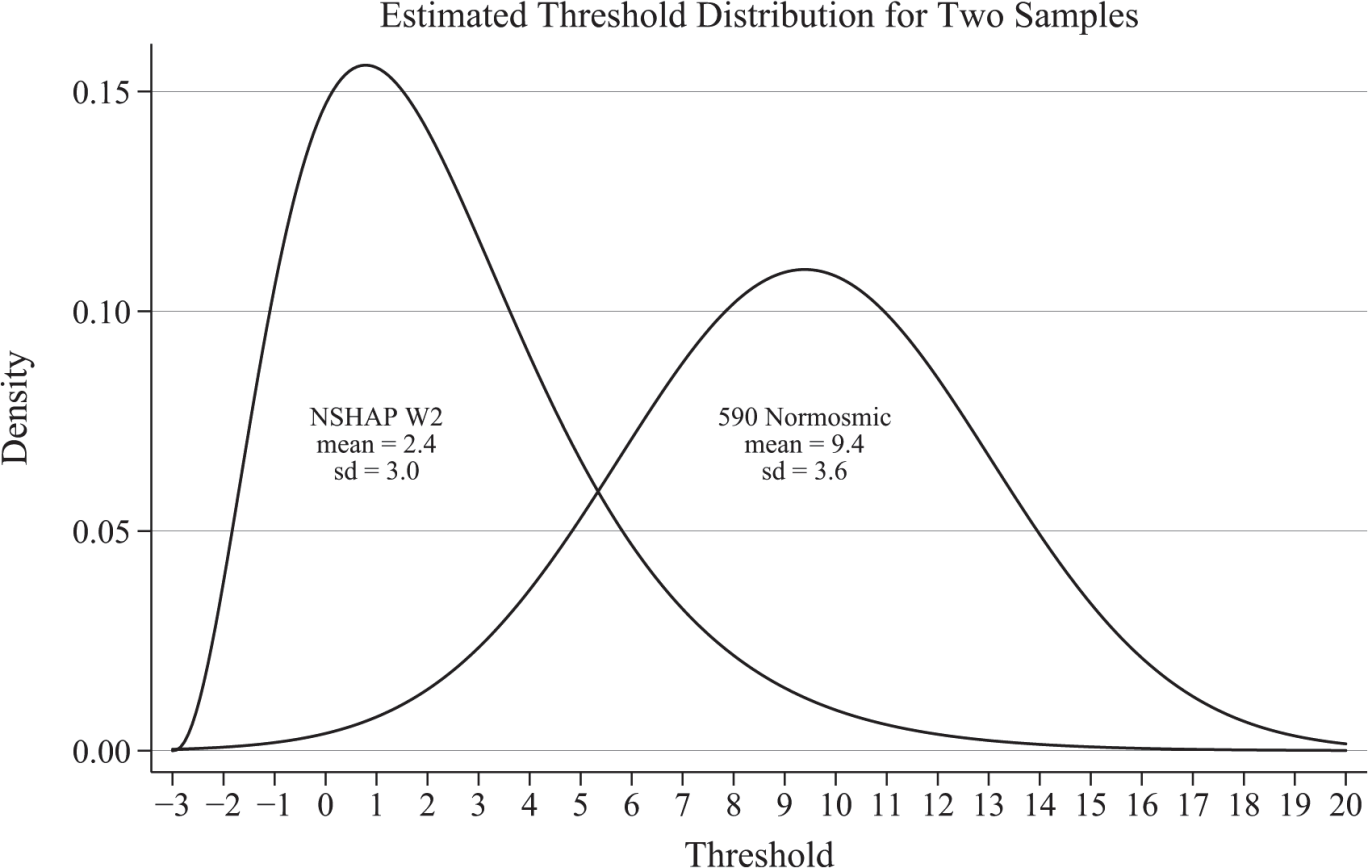
Distribution of Thresholds for Normosmics and NSHAP. Threshold distributions used for simulations, based on model-estimated distributions for the 590 normosmic sample and the NSHAP Wave 2 sample (n = 2,207) of older adults. The means and standard deviations for both samples are as estimated by the IRT model; the Normal distribution for the NSHAP sample has been replaced by a gamma distribution with identical mean and standard deviation, to more closely approximate the likely distribution of thresholds in the population.

Given that thresholds of 4% *n*-butanol or greater are often considered indicative of functional anosmia, we substituted a three-parameter gamma distribution with shape parameter 3.35, scale parameter 1.61 and location parameter-3 (Fig. 3). This distribution has the same mean and standard deviation as the estimated Normal distribution, but its positively-skewed shape is more likely to match the distribution of actual thresholds in this population. In particular, according to this distribution, 9% of the population has a threshold of 16% *n*-butanol or greater, only 1% has a threshold of 32% or greater, and none has a threshold of 64% or greater. The estimated slope parameter of 0.76 was used to simulate individual responses. A sample size of 2,000 respondents was used to approximate the NSHAP sample, and as before, 500 replications per condition were performed.

The mean correlation between the model-estimated thresholds from data collected using the NSHAP protocol and the true thresholds was 0.75, yielding an estimated reliability for the NSHAP protocol among older adults of 0.56 (Table 3, row 3). Surprisingly, the reliability was only slightly higher when administering the full 16 dilutions used by Lötsch et al. (0.65; Table 3, row 1). This is due to the fact that many of those dilutions are far from the center of the threshold distribution for older adults. However, reliabilities for other possible 6-dilution protocols were considerably lower; for example, 6 dilutions chosen evenly from across the full range of standard dilutions (a plausible approach in the absence of any information about the study population) yielded a reliability of only 0.36.

**Table 3:** Reliability of constant stimuli protocols based on simulated administration to the NSHAP Wave 2 distribution, n = 2,000 respondents with 500 replications.

	Number of Stimuli	16	6
**Evenly Distributed**	Dilutions Administered	1–16	2, 5, 8, 9, 12, 15
	Mean Reliability^a^	0.648	0.358
	5%, 50%, 95%	0.622, 0.648, 0.671	0.321, 0.361, 0.395
	% of Convergence Failures	0.0%	9.2%
**Centered**	Dilutions Administered	NA^b^	8–13
	Mean Reliability		0.125
	5%, 50%, 95%		0.065, 0.126, 0.172
	% of Convergence Failures		60.2%
**NSHAP Protocol**	Dilutions Administered	NA	0, 1, 2, 4, 5, 6
	Mean Reliability		0.558
	5%, 50%, 95%		0.534, 0.558, 0.581
	% of Convergence Failures		0.0%
**Shifted**	Dilutions Administered	NA	0, 2, 4, 6, 8, 10
	Mean Reliability		0.486
	5%, 50%, 95%		0.458, 0.486, 0.515
	% of Convergence Failures		0.0%

^a^All reliabilities have a standard error ≤ 0.001

^b^NA = not applicable.

**Fig 4:**
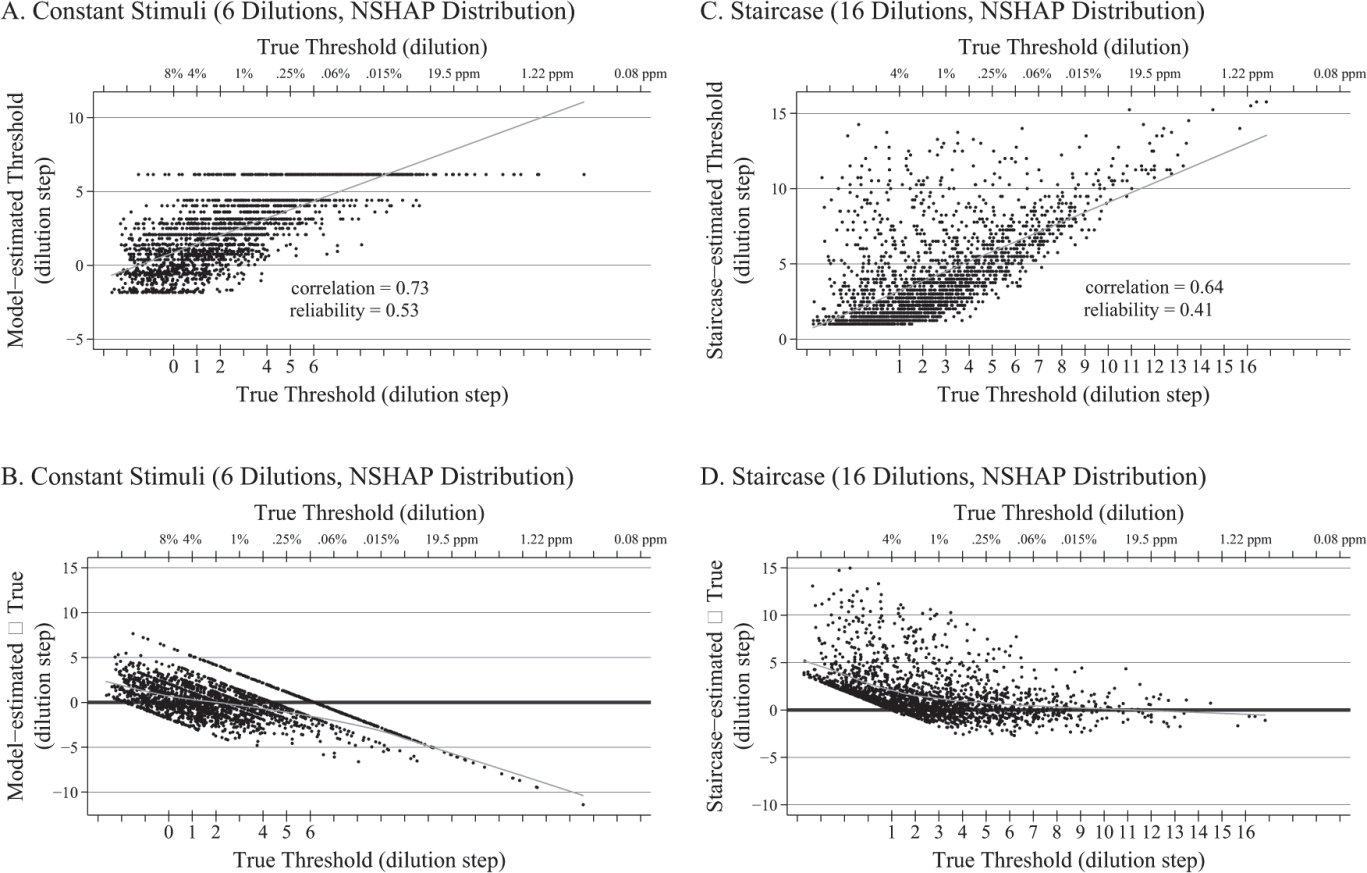
Scatter and Bland-Altman Plots for NSHAP. A. Scatter plot (plus regression line) of the relationship between the model-estimated thresholds and the true thresholds for a single replication of the 6-dilution NSHAP constant stimuli protocol (dilutions administered are noted on the lower x-axis), with thresholds drawn from a gamma distribution with mean and variance matching those estimated from the NSHAP sample (n = 2,000). B. Corresponding Bland-Altman plot with LOWESS smoother of the NSHAP 6-dilution protocol, with thresholds drawn from a gamma distribution with mean and variance matching those estimated from the NSHAP sample (n = 2,000). C. Scatter plot (plus regression line) of the relationship between the staircase-estimated thresholds and the true thresholds for a single replication of the 16-dilution staircase design, with thresholds drawn from a gamma distribution with mean and variance matching those estimated from the NSHAP sample (n = 2,000). D. Corresponding Bland-Altman plot with LOWESS smoother of the 16-dilution staircase design, with thresholds drawn from a gamma distribution with mean and variance matching those estimated from the NSHAP sample (n = 2,000).

A plot showing the relationship of the model-estimated thresholds for the NSHAP 6-dilution constant stimuli protocol and the true thresholds for a single replication is shown in Fig. 4A, and a corresponding Bland-Altman plot with LOWESS smoother is shown in Fig. 4B. The model-estimated thresholds are unconditionally unbiased (that is, for the sample as a whole), but as with the evenly distributed 6-dilution design administered to normosmics (Fig. 2, Panel D), the conditional bias increases toward the tails of the distribution—especially at the weak end, due to the lack of pens with weak concentrations. As a result, the method underestimates the ability of the best smellers, though these represent a relatively small proportion of the population of older adults.

Note that the apparent gap in Fig. 4A between a model-estimated threshold of 6.1 and the next highest estimated threshold of 4.4 (as well as the corresponding gap in Fig. 4B) is an artifact of the inherent discreteness in using only 6 stimuli, yielding a total of only 64 possible response patterns. Although the 63 response patterns corresponding to 0–5 correct items yield estimated thresholds which overlap (for example, a response pattern with 5 dilutions correctly detected may yield an estimated threshold similar to a response pattern with only 4 dilutions correctly detected, depending on where the correct responses appear), with only 6 stimuli there are no response patterns to fill the gap between 6 correct and the next highest pattern (5 correct responses with one error at the weakest dilution).

Finally, when the full staircase method (with 16 dilutions) is administered via simulation to the NSHAP distribution and scored in the standard way (average of the last four reversals), the reliability is actually *lower* than for the NSHAP 6-dilution protocol (reliability = 0.41; Fig. 4, Panel C), and the conditional bias at the low end of the threshold distribution where the odorant concentrations are strongest is greater (Fig. 4, Panel D). This results from the limitation in the standard scoring method identified above, by which two or more erroneous reversals early on among the weaker dilutions can lead to respondents with relatively poor olfaction receiving relatively high threshold estimates representative of better smellers (Fig. 1, Panel B)—a tendency that is exacerbated in the NSHAP sample due to the high proportion with poor olfaction. It is important to recognize that this is a limitation of the scoring procedure only, and that apart from the time and training required, there is no problem in principle with using the staircase method to collect data from a sample of older adults (although the addition of one or more pens with stronger concentrations would increase the ability to discriminate among the poorest smellers). However, once the data have been collected, using the IRT model to estimate individual thresholds will reduce bias and increase precision.

### Public Availability of Data and Software

The method described here for estimating the performance of a specific staircase or constant stimuli protocol intended to assess olfactory threshold can be easily applied by researchers who wish to evaluate or design a specific assessment (not limited to olfaction) for their own purposes. As illustrated here, the first step in designing a new protocol is to acquire information about the threshold distribution in the target population. Hummel et al. [13] provide normative data for *n*-butanol threshold, including means, standard deviations and percentiles for threshold distribution by age and gender. The NSHAP Wave 2 data described here are publicly available through the National Archive of Computerized Data on Aging (NACDA, https://www.icpsr.umich.edu/icpsrweb/NACDA/) [30], and provide data for the general population of home-dwelling, older adults up through age 90. Even if one is focusing on a unique or narrowly defined subpopulation for which he or she already has data, consulting publicly-available reports and data sources is encouraged, especially if one’s own data are from a small sample. Although data on *n*-butanol threshold may be informative in planning a threshold testing protocol to be used with a different odorant, potential differences between odors in sensitivity should be considered.

The software used here to simulate both the staircase and constant stimuli protocols is available at http://rcg-software.uchicago.edu/stata in a package called olfactsim for use with Stata, and includes a description of the steps necessary to evaluate a new or existing protocol [31]. Given a pre-specified threshold distribution, this package is able to simulate the administration of both the staircase and constant stimuli protocols, each with an arbitrary set of dilutions (repetition of one or more dilutions when simulating the constant stimuli protocol is also possible). In addition to exploring several designs of the maximum length that can be administered to actual subjects, we also recommend trying several threshold distributions with mean and variance varied around the values estimated for the target population. Doing so facilitates an examination of the sensitivity of the results to miss-specifying the population distribution.

## Discussion

We have shown here how computer simulation can be used to evaluate the measurement properties of tests of olfactory threshold without the cost or time required to conduct a study with human subjects. This method is capable of reproducing estimates of reliability obtained from human subjects studies, with the possible exception of a relatively small component of variability due to short-term variation within subjects themselves and/or in the physical administration of the test (due to slight variations in the pens or the way in which they are administered). This additional variability could be estimated by comparing the test-retest reliability estimated from human subjects studies to the reliability estimated from simulation, and incorporated into the reliability estimates generated from the latter, if desired.

Unlike the staircase method, assessment based on the method of constant stimuli requires using an IRT model to obtain individual threshold estimates. This model can also be fit to data collected using the staircase method to obtain less biased and more precise threshold estimates than the standard scoring method. Although such a model can be fit separately to the data for each subject if the number of stimuli is large, a random effects version of the model must be used when the number of stimuli is small.

Moreover, the shrinkage estimates of threshold—obtained either by using empirical Bayes methods following maximum likelihood estimation of a random effects model, as done here, or by using a hierarchical Bayesian model—yield threshold estimates which, when used as a covariate in a subsequent regression model, eliminate attenuation bias due to measurement error. Still, for those researchers who want an immediate measure of olfactory performance without having to fit a statistical model, the correlation between the model-estimated thresholds (with multiple imputation for non-response) and the number of correct responses (0–6, treating don’t know and refused as incorrect) in the NSHAP dataset is 0.92. Thus, the latter may be adequate in certain cases, such as when the objective is merely to demonstrate a relationship between olfaction and another variable.

There are ways in which the IRT model used here might be extended. For example, we do not consider a learning effect, though estimating such an effect would require that the order of presentation of dilutions be varied across subjects, and any learning effect is likely to affect only the first few presentations. More important, perhaps, is the fact that both the IRT model and our simulations assume that everyone has an actual threshold value (a dilution which they are able to detect with probability $\frac{2}{3}$), therefore excluding the possibility of subjects who are truly anosmic (unable to detect the odor at any concentration). A mixture model [32] in which a proportion of subjects (to be estimated from the data) are truly anosmic may match reality more closely—especially among older adults—however estimating such a model would likely require several additional dilutions, possibly with repetition, at the low end (high concentration).

To our knowledge, our results show for the first time that home-dwelling older adults have substantially worse olfactory sensitivity, a finding that has important implications for the health and safety of older adults and the resulting public health burden. Further, these results confirm the value of the constant stimuli method for obtaining valid and reliable estimates of olfactory threshold in cases where restrictions on the length and/or complexity of the test preclude administering the staircase method. We have demonstrated that even with only 6 dilutions each presented once, reliabilities in the range of 0.56–0.67 can be achieved, thereby extending the possibility of olfaction threshold measurement to a much wider range of research settings. When possible, however, higher reliability is generally desirable, and may be achieved even with only 6 dilutions by presenting each dilution twice. Finally, it should be noted that we did not examine the reliability of model-based estimates using data collected with the staircase method, nor other areas in which the staircase method may have an advantage due to its dynamic presentation of dilutions. For example, the staircase method has successfully been employed using the Sniffin’ Sticks in a wide range of clinical research [33–35]. Thus, when possible, researchers may still wish to use this method, though ideally with model-based threshold estimates.

Doty et al. [36] called attention to the importance of reliability in olfaction assessments, focusing on the relationship between reliability and the number of stimuli administered. The results reported here underscore the critical importance not only of the total number of stimuli, but also of the choice of dilutions relative to the threshold distribution of the population. This is evident in the fact that for the NSHAP population, 6 dilutions spread evenly across the range of dilutions yielded considerably lower reliability than 6 dilutions concentrated under the largest area of the NSHAP threshold distribution (0.36 versus 0.56). Further, 6 dilutions picked from the center of the dilution range with only minimal coverage of one tail of the NSHAP distribution yielded the lowest reliability reported (0.13). Lastly, even 16 dilutions when spread across the full range only increased the reliability to 0.65. Thus, researchers designing their own measurement protocol should consider carefully the olfactory ability of the population to which it will be administered.

In this investigation we have focused on overall reliability, since a main goal of the NSHAP study is to examine the relationship between olfactory threshold and other variables. However, those wishing to use olfactory threshold measurement for other purposes (for example, in a clinical setting) will probably want to focus on other performance aspects, such as bias for individual threshold measurements and the sensitivity and specificity associated with specific cutoffs. The computer simulation method described here could be used equally well to examine these quantities.
